# Pharmacology, Therapeutics and Materia Medica

**Published:** 1886-01

**Authors:** 


### Book Reviews.

---

PHARMACOLOGY, THERAPEUTICS AND MATERIA MEDICA. By Lander Brunton, M. D., D. Sc., F. R. S. Adapted to the United States Pharmacopœia by Francis H. Williams, M. D. Philadelphia: Lee Brothers. & Co. 1885. 8vo., pp. 1035.

This work, as the writer says in the preface, is the result of fifteen years of thought, observation and experimentation, and in reading the book we were prepared to come across something striking in the way of discovery, or application of remedial agents, but there was nothing startling in this line. The action of drugs, according to eminent pharmacologists and physiologists, receives confirmation, and is presented together with interesting and valuable details in a most practical form.

The first section is devoted to the subject of pharmacology and therapeutics. The physiological action of drugs on various parts of the organism is discussed, *i. e.*, the manner in which the part is affected by the drug. Then follows a list of substances that affect the part or organ in the manner described. The remaining sections of the book are devoted to general pharmacy and materia medica.

In discussing the action of drugs on the digestive system, the author makes a striking statement as to the effect of a small dose of opium. It is well known that opium may relieve constipation when due to localized irritation, the result of some obstruction in the bowels, but when we are told that one-half a drop of the tincture of opium in a case of ovarian disturbance "acted as a brisk purgative," we are reminded of infinitesimals and diluted nothings. Constipation may be due to increased action of the inhibiitory fibres of the splanchnic or to some paralyzed condition of the accelerator fibres of the vagus. The balance of nerve influence may be disturbed to such a slight degree that a small dose of a drug is sufficient to restore the equilibrium. But the effect of one-half a drop of the tincture of opium seems to us rather imaginary, especially in "delicate women of a nervous temperament suffering from ovarian pain." In such cases we have already seen an hypnotic effect follow the hypodermic injection of *aqua pura.*

The writer gives Dr. Crawford W. Long, of Athens, Georgia, the credit of being the first to use ether in surgical operations. It is a noteworthy and specially commendable feature of the book that the arrangement of substances is according to scientific methods. In inorganic materia medica the substances are classed according to a chemical principle, and in vegetable materia medica drugs are placed together under their appropriate botanical class and division.

In the preface the writer says that the work "is intended not only as a text-book for students, but also for the use of practitioners for the purposes of reference." As a text-book it ought to be a success, as it contains in concise and systematic form the knowledge necessary for the student, avoiding general discussion and those subjects which are to a great extent *sub judice.* As a reference book, especially on the subject of materia medica, while it contains much that is valuable, it is not sufficiently comprehensive in the description of drugs and medicinal preparations. H. W.

